# Long-Term Outcome of Eyes with Vitrectomy for Submacular and/or Vitreous Hemorrhage in Neovascular Age-Related Macular Degeneration

**DOI:** 10.1155/2021/2963822

**Published:** 2021-11-02

**Authors:** Setsuko Kawakami, Yoshihiro Wakabayashi, Kazuhiko Umazume, Yoshihiko Usui, Daisuke Muramatsu, Tsuyoshi Agawa, Kaori Yamamoto, Hiroshi Goto

**Affiliations:** Department of Ophthalmology, Tokyo Medical University, Tokyo 160-0023, Japan

## Abstract

**Purpose:**

To study long-term clinical outcomes in patients with submacular hemorrhage (SMH) and/or vitreous hemorrhage (VH) associated with neovascular age-related macular degeneration (nAMD) and the real-world clinical situation of adding anti-VEGF therapy after pars plana vitrectomy (PPV).

**Methods:**

In this retrospective case series, 25 eyes with SMH and/or VH associated with nAMD were treated by PPV and followed up for at least 24 months. When exudative changes were unresolved or recurred after PPV, additional intravitreal anti-VEGF therapy was given.

**Results:**

The reasons for performing PPV were SMH (8 eyes) and VH (17 eyes) associated with nAMD. Mean best-corrected visual acuity (BCVA) of eyes with SMH improved significantly at 6 months (*P* < 0.01) and 12 months (*P* < 0.05) after PPV. Mean BCVA of eyes with VH improved at 1, 3, 6, 12, 18, and 24 months (*P* < 0.01) and at the final visit (*P* < 0.05). Post-PPV anti-VEGF therapy was initiated in 6 of 8 (75.0%) eyes with SMH and 7 of 17 (47.1%) eyes with VH. Of the 13 eyes given anti-VEGF therapy after PPV, 11 eyes had anti-VEGF therapy initiated within 10 months after surgery. Dry macula rate after PPV was 50.0% in SMH and 70.6% in VH.

**Conclusions:**

BCVA improved in eyes with SMH at 6 and 12 months after PPV, and the BCVA was maintained until the end of the study. BCVA improved significantly in eyes with VH at all time points after PPV. In eyes undergoing PPV for nAMD, recurrence of exudative changes after 11 months from the initial PPV was rare.

## 1. Introduction

Antivascular endothelial growth factor (VEGF) therapy is the first-line therapy for neovascular age-related macular degeneration (nAMD), and sometimes pars plana vitrectomy (PPV) is required in highly active cases with submacular hemorrhage (SMH) or vitreous hemorrhage (VH). As treatments for SMH, both “nonvitrectomy” [1–3] and “vitrectomy” methods [4–6] have been reported. Compared to vitrectomy, nonvitrectomy treatment is easier to administer and facilitates early treatment. On the other hand, vitrectomy is likely to remove fully SMH, especially preexisting VH or thick SMH [7]. While PPV may reduce the activity of nAMD [8, 9], unresolved or recurrent exudative changes may require addition of anti-VEGF therapy even after PPV. However, the half-life of anti-VEGF agent is shortened in vitrectomized eyes [10–12], and there is concern that the effect may be diminished faster compared to nonvitrectomized eyes.

The aim of this study was to estimate the prognosis of nAMD with SMH or VH necessitating PPV and to examine appropriate management after PPV. We also investigated the long-term outcome and the real-world clinical situation of additional anti-VEGF therapy after PPV.

## 2. Materials and Methods

This study was a retrospective case series of consecutive patients with nAMD complicated by SMH or VH, who were treated with PPV and followed up for at least 24 months after PPV at the Department of Ophthalmology of Tokyo Medical University Hospital between April 2009 and March 2017. The study adhered to the tenets of the Declaration of Helsinki and was approved by the institutional review board. All subjects provided written informed consent for the treatment and for this study. Inclusion criteria were nAMD with thick SMH of at least 1.5 disc diameter involving the fovea or VH. Exclusion criteria were other concurrent intraocular diseases.

The patients were examined for best-corrected visual acuity (BCVA), intraocular pressure, slit-lamp biomicroscopy, indirect ophthalmoscopy, OCT examination, and fluorescein and indocyanine green angiography before the initial PPV (baseline). Subtypes of nAMD, namely, polypoidal choroidal vasculopathy (PCV), retinal angiomatous proliferation (RAP), and typical AMD (tAMD), were diagnosed by fundus examination, optical coherence tomography (OCT), and fluorescein and indocyanine green angiography as reported previously [13–18]. However, as the presence of hemorrhage resulted in poor image quality, postoperative imaging was performed if preoperative evaluation was not possible.

The surgical procedures were as follows. In all eyes, a standard 3-port 25-gauge PPV was performed, and posterior vitreous detachment was created if not already present. At the time of PPV, phacoemulsification, aspiration and intraocular lens implantation (PEA-IOL), injection of 32,000 IU/0.2 ml of tissue plasminogen activator (tPA) (Monteplase; Eizai Co, Tokyo, Japan) solution into the subretinal space using a 38-gauge flexible cannula (Alcon), and intravitreal injection of anti-VEGF agents were performed as needed (discretion of the operator). Intravitreal bevacizumab (IVB) was used for anti-VEGF therapy during PPV. Fluid-air exchange and tamponade with air, SF6, or silicone oil was also conducted when necessary.

After the initial PPV, the patients were examined every month for BCVA, intraocular pressure, slit-lamp biomicroscopy, indirect ophthalmoscopy, and OCT examination. From 6 months or more after PPV, these examinations were performed every 2–4 months in less active cases. Intravitreal injection of anti-VEGF agents was performed after PPV for unresolved or recurred exudative changes detected using an ophthalmoscope or by OCT. In some cases, the treat and extend (TAE) regimen [19, 20] was used for subsequent injections depending on the activity of nAMD while receiving anti-VEGF therapy. In the case of TAE, the injection interval was extended or shortened to a maximum of 16 weeks with adjustment every 2 or 4 weeks. For some cases where the recurrence period was fixed, fixed dosing was used.

Visual acuity was measured using decimal visual acuity chart and converted to logarithm of minimal angle resolution (logMAR). Counting fingers, hand motion, and light perception were also converted to logMAR as reported previously [21, 22]. BCVA improvement or worsening was defined as improvement or worsening of more than 0.3 logMAR. Statistical analysis was performed using MedCalc Statistical Software version 16.2.0 (MedCalc Software Bvba, Ostend, Belgium; https://www.medcalc.org; 2016). Wilcoxon signed-rank test was used for comparison of preoperative and postoperative visual acuity, and Mann–Whitney's *U*-test was used to compare two groups. The correlation between the amount of change in visual acuity and each factor was evaluated using the Spearman's correlation coefficient by rank test. *P* values less than 0.05 were statistically significant.

## 3. Results

In this study, 25 eyes of 25 patients (9 women and 16 men) were included. All patients were followed up for at least 24 months after the initial PPV. Baseline characteristics of the patients are listed in Table 1.

The reasons for performing PPV were SMH (8 eyes) and VH (17 eyes) associated with nAMD. The mean age was 73.8 ± 13.2 (range 48–89) years at the initial PPV in eyes with SMH, 77.3 ± 7.1 (67–89) years in eyes with VH. As for subtypes of nAMD, PCV, RAP, and tAMD were diagnosed, respectively, in 7, 0, and 1 eyes with SMH, and in 11, 2, and 4 eyes with VH. The mean greatest diameter of the SMH was 4.0 ± 1.6 (range 2.0–6.7) disc diameters. The mean BCVA was 0.96 ± 0.65 logMAR (range 0.01–0.7, decimal) in eyes with SMH and 1.82 ± 0.67 logMAR (range LP (+)−0.6) in eyes with VH. The mean time from onset to initial PPV was 9.3 ± 4.6 (range 4–17) days in eyes with SMH and 45.9 ± 40.0 (range, 13–142) days in eyes with VH (except 4 eyes with unknown duration).

Treatment before initial PPV, additional procedure during initial PPV, and treatment after initial PPV are shown in Table 2. Before the initial PPV, 2 eyes with SMH and 8 eyes with VH had intravitreal injection of anti-VEGF, 3 eyes with VH had photodynamic therapy (PDT), and 1 eye with VH had photocoagulation (PC) for extrafoveal CNV. During operation, tPA was injected into the subretinal space in 8 eyes with SMH and in 5 eyes with VH. After the initial PPV, 6 eyes with SMH and 7 eyes with VH received anti-VEGF therapy. One eye with VH underwent PDT and subsequently received anti-VEGF therapy, and two eyes with VH underwent PDT after initiation of anti-VEGF therapy. Three eyes underwent PC for extrafoveal CNV after PPV, one eye underwent PC after anti-VEGF induction, and the other two eyes underwent PC only. Two eyes with SMH underwent repeat PPV for postoperative macular hole and postoperative proliferative vitreoretinopathy, and one eye with VH for silicone oil removal. One eye with VH had second PPV for recurrent VH and third PPV plus scleral buckling for retinal detachment.

**Figure 1:**
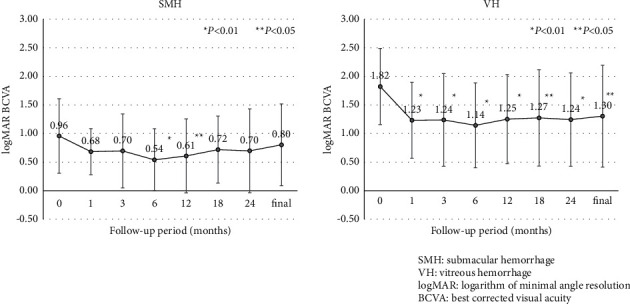
Mean best-corrected visual acuity (BCVA) (logMAR) in an eye with (a) SMH and (b) VH.

Figure 1 shows the mean BCVA of SMH (*n* = 8) and VH (*n* = 17) eyes that underwent PPV. The mean BCVA (logMAR) at baseline, 1, 3, 6, 12, 18, and 24 months after initial PPV and at final examination were, respectively, 0.96, 0.68, 0.70, 0.54, 0.61, 0.72, 0.70, and 0.80 in eyes with SMH; and 1.82, 1.23, 1.24, 1.14, 1.25, 1.27, 1.24, and 1.30 in eyes of VH. Significant improvement compared to preoperative BCVA (0 month) was observed at 6 months (*P* < 0.01) and 12 months (*P* < 0.05) after vitrectomy in eyes with SMH; and at 1, 3, 6, 12, 24 months (*P* < 0.01), 18 months, and at the final visit (*P* < 0.05) in eyes with VH.

In both SMH and VH eyes, there was no significant difference in BCVA at any time compared to 1 month after initial PPV (*P* > 0.05). At the final visit, BCVA improved (more than 0.3 logMAR compared to before PPV) in 4 (50.0%) and worsened in 1 (12.5%) of 8 eyes with SMH and improved in 11 (64.7%) and worsened in 3 (17.6%) of 17 eyes with VH. The causes of vision loss were fibrosis in eyes with SMH and chronic macular edema, fibrosis, and organized SMH in eyes with VH. In eyes with SMH, there was no correlation between preoperative factors (age, period from onset of SMH or VH to PPV, greatest diameter of SMH, and baseline BCVA) and the amount of BCVA change. In eyes with VH, there were no significant differences in the amount of BCVA change by gender, PCV or non-PCV, history of anti-VEGF before PPV, or use of tPA during PPV.

**Figure 2:**
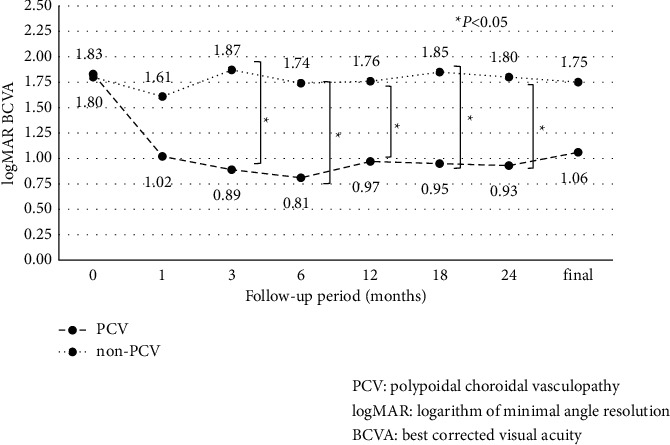
A comparison of best-corrected visual acuity (BCVA) between PCV and non-PCV in eyes with VH.

A comparison of visual acuity between PCV and non-PCV in eyes with VH showed that visual outcome was significantly better in PCV eyes than in non-PCV at 3, 6, 12, 18, and 24 months after the initial PPV, although there was no significant difference at the final observation (Figure 2). The mean follow-up period was 52.6 ± 20.5 months for PCV eyes and 55.7 ± 24.8 months for non-PCV eyes.

Table 3 shows anti-VEGF therapy before and after PPV in the SMH group and VH group. 6 of 8 (75.0%) eyes with SMH and 7 of 17 (41.2%) eyes with VH underwent anti-VEGF therapy after PPV. 2 of 6 (33.3%) eyes with SMH and 3 of 7 (42.9%) eyes with VH that underwent anti-VEGF therapy after PPV maintained dry macula for more than 1 year after the last injection. Thus, in all the subjects analyzed, dry macula was achieved in 4 of 8 (50.0%) eyes with SMH and 12 of 17 (70.6%) eyes with VH. The average time from the initial PPV to initiation of anti-VEGF therapy was 6.5 ± 9.3 (range 1–25) months in eyes with SMH and 8.4 ± 11.1 (range 1–32) months in eyes with VH.

**Figure 3:**
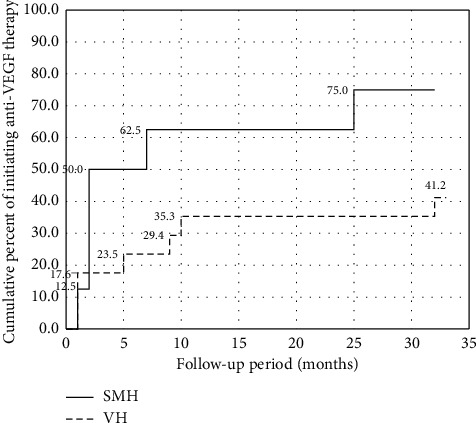
Cumulative percent of anti-VEGF therapy initiation.

The cumulative percentage of anti-VEGF therapy initiation during follow-up is shown in Figure 3. The rate of anti-VEGF therapy initiation after PPV was 12.5% after 1 month, 50.0% after 2 months, 62.5% after 7 months, and 75.0% after 25 months in 8 eyes with SMH; 17.6% after 1 month, 23.5% after 5 months, 29.4% after 9 months, 35.3% after 10 months, and 41.2% after 32 months in 17 eyes with VH. Of the 13 eyes in which anti-VEGF therapy was administered after PPV, 11 eyes had anti-VEGF initiated within 10 months after surgery. Thereafter, anti-VEGF was initiated only sporadically at 25 and 32 months after PPV due to recurrence of exudative changes.

Table 4 lists the ocular findings, treatment contents, and outcome of all the individual subjects analyzed in this study. The clinical findings of individual patients were reviewed. Among patients with SMH who underwent PPV, 4 of 6 eyes given anti-VEGF therapy after PPV had improvement of final BCVA by logMAR 0.3 or more. In one eye with fibrosis, BCVA decreased even after initiation of anti-VEGF, and only one injection was administered. In two eyes with dry macula without anti-VEGF therapy, BCVA was unchanged.

Regarding the status of anti-VEGF therapy before and after PPV, among 6 eyes with SMH that received anti-VEGF therapy after PPV, 4 eyes had no treatment before PPV and the remaining 2 eyes had a history of anti-VEGF therapy before PPV (for duration of 2.78 months before PPV and 45.00 months after PPV in one, 13.33 months before PPV and 4.67 months after PPV in the other). Among 7 eyes with VH that received anti-VEGF therapy after PPV, 4 eyes had a history of anti-VEGF before PPV. Of these 4 eyes, 2 eyes had clear details of their anti-VEGF treatment history prior to PPV and no other treatment interventions. In the 2 eyes, the durations of pre- and post-PPV anti-VEGF therapy were, respectively, 1.69 and 3.13 months and 1.17 and 22.0 months.

## 4. Discussion

Although there are several reports of using PPV and subretinal tPA for treating SMH associated with nAMD, there are few reports of long-term follow-up of cases requiring post-PPV anti-VEGF therapy. Kimura et al. [23] reported that, in patients with SMH associated with PCV, BCVA improved significantly after 1 month (*p*=0.001), with an improvement of 0.39 logMAR from baseline to the last follow-up (*p* < 0.01) (33 ± 14 months). In our study, BCVA showed significant improvement from 6 to 12 months after the first PPV compared to baseline. At the final visit (average follow-up 32.6 ± 6.7 months), BCVA improved by 0.16 logMAR compared to baseline, but did not maintain a significant improvement.

The rate of anti-VEGF therapy initiation after PPV in eyes with SMH was reported to be 38.6% (caused by nAMD) for a mean follow-up period of 15.3 months by Chang et al. [4] and 91% (caused by PCV) for a mean follow-up period of 33 months by Kimura et al. [23]. We initiated anti-VEGF therapy at a rate of 75.0% for a mean follow-up period of 32.6 months. The rate of initiation of anti-VEGF therapy varies among studies because follow-up period and the conditions for starting anti-VEGF therapy were different. The use of postoperative anti-VEGF medications is decided by the treating physician, and the initiation criteria are not clear [4]. Our group and Kimura et al. [23] started administration when exudative or hemorrhagic changes (such as accumulation of subretinal fluid) and recurrence of SMH were observed after PPV. Using this strategy, the rate of anti-VEGF therapy initiation appears to be relatively high.

According to previous reports, the frequency of intravitreal anti-VEGF injections after PPV was 4.2 times per year with a PRN regimen [23] and 3.1 times per year with a regimen of two monthly doses changing to PRN in all patients after PPV [24]. In our study, the frequency was 3.4 times for a basically PRN regimen, although some cases of proactive protocols such as TAE and fix regimens were included. Therefore, it is possible that the number of doses administered was higher than the number of recurrences.

At the final visit, BCVA improved in 4 eyes and worsened in 1 eye with SMH. All the 4 eyes with improved vision had received multiple anti-VEGF injections after PPV. BCVA decreased even after the initiation of anti-VEGF in 1 eye with fibrosis, and only one injection was administered in this eye. In 2 eyes that had dry macula without anti-VEGF, BCVA was unchanged. By reviewing the clinical course of individual patients, there was an impression that increasing the frequency of anti-VEGF injections was not a factor associated with poor visual outcome. During PPV, tPA was used in all the eyes with SMH. Although tPA was used during PPV in some of the VH patients, there was no significant difference in the amount of BCVA change with or without tPA. Previous reports have suggested the possibility of tPA toxicity in addition to damage to photoreceptor cells and retinal pigment epithelium due to hematologic toxicity in SMH eyes [25, 26], although it remains unclear whether the use of tPA limited the improvement of visual acuity in this study. Further investigation is needed to examine the effects of tPA usage.

In eyes with VH, average BCVA improved significantly at all time points compared to baseline. The improvement of BCVA in VH eyes was greater than in SMH eyes because removal of VH by PPV per se improved BCVA. According to reports of PPV for nAMD with breakthrough VH [27–31], improvement of BCVA logMAR from baseline to the last follow-up was 0.82–1.22 (follow-up period: 12.7–25.2 months) in eyes with PCV and 0.00–0.92 (follow-up period: 17.0–29.1 months) in eyes with AMD (non-PCV). In our study, improvement of BCVA logMAR from the baseline to last follow-up was 0.77 (follow-up period: 52.6 months) in PCV eyes and 0.05 (follow-up period: 55.7 months) in non-PCV eyes. PCV eyes had significantly better BCVA than non-PCV eyes up to 24 months, and the results were generally consistent with previous reports, considering the follow-up periods. However, the difference between PCV and non-PCV seemed to decrease with longer follow-up. It is difficult to investigate this phenomenon because of the difficulty to evaluate the fundus before PPV in VH eyes, and the fundus findings are modified by PPV.

The percentage of eyes receiving anti-VEGF therapy before PPV tended to be higher in eyes with VH (47.1%) than in eyes with SMH (25.0%), while the rate of anti-VEGF therapy initiation after PPV tended to be lower in eyes with VH (41.2%) than in eyes with SMH (75.0%). It is difficult to evaluate whether PPV has reduced the recurrence of exudative changes in eyes with SMH because nAMD treatment was started at the time of initial PPV in many eyes with SMH. In contrast, pre-PPV treatment for nAMD was present in nearly one-half of the eyes with VH. We examined the durations of anti-VEGF therapy before and after PPV in 2 eyes of VH with a clear history of anti-VEGF therapy before surgery and no other treatment interventions and showed that the duration of anti-VEGF was prolonged after PPV. Although we were only able to compare a small number of cases, the duration between anti-VEGF therapy after PPV tended to be longer in the comparable cases, suggesting that the CNV activity of nAMD tends to decrease after PPV, as reported by Ikeda et al. [8] and Sakamoto et al. [9]. There are few reports on long-term postoperative observations of eyes with VH and on the use of anti-VEGF when needed. Regarding the changes in anti-VEGF therapy before and after PPV for eyes with VH, Kim et al. [32] reported that the mean duration of anti-VEGF administration was significantly prolonged from 4.53 months before PPV to 27.64 months after PPV. In addition, initiation of anti-VEGF therapy decreased from 11 months after the initial PPV, suggesting that new recurrence of exudative changes may be inhibited after a long period of time following PPV.

There is little evidence in the literature on the effect of anti-VEGF therapy on nAMD in vitrectomized human eyes. Hahn [33] presented a case of successful treatment with aflibercept following single bevacizumab failure in a patient with recurrent choroidal neovascularization following prior macular translocation vitrectomy surgery for AMD. Jung et al. [34] reported a case series of 4 patients with AMD who were previously vitrectomized for macular pucker or macular hole. In their study, treatment with aflibercept was effective in controlling AMD. Because of faster clearance of anti-VEGF drug from vitrectomized eyes, injection of anti-VEGF drug in vitrectomized eyes is expected to be less efficient than in nonvitrectomized eyes [10–12]. On the other hand, there are also reports that the intraocular pharmacokinetic profile of anti-VEGF drug in vitrectomized eyes is similar to that in non-vitrectomized eyes [35]. In this study, we did not examine the effect of anti-VEGF therapy without PPV in SMH eyes and in VH eyes. Therefore, it is unclear whether the effect of anti-VEGF therapy is attenuated in vitrectomized eyes. However, according to the clinical course of individual patients, visual acuity was improved or maintained while on anti-VEGF therapy after PPV, suggesting that anti-VEGF therapy may be effective in vitrectomized eyes.

There are several limitations in this study. First, due to the small number of cases, this study lacks statistical power to detect significant differences. Second, in some cases, it was difficult to accurately assess the status of nAMD before PPV. Third, anti-VEGF therapy after PPV was generally used as reactive treatment, but was also used as proactive treatment in some patients. Therefore, the number of anti-VEGF injections did not accurately reflect the status of exudative changes.

In conclusion, BCVA improved in eyes with SMH at 6 and 12 months after PPV and was maintained until the last visit. BCVA improved significantly in eyes with VH at all time points after PPV. In eyes undergoing PPV for nAMD, recurrence of exudative changes after 11 months of initial PPV was rare.

## Figures and Tables

**Table 1:** Baseline characteristics of the patients.

Characteristics	SMH	VH
Number of patients/eyes	8/8	17/17
Gender ratio: male/female	4/4	12/5
Average age, years	73.8 ± 13.2 (48–89)	77.3 ± 7.1 (67–89)
Lens status: phakic/IOL	7/1	11/6
Subtype of nAMD, number of eyes		
** **PCV/RAP/tAMD	7/0/1	11/2/4
** **PCV/non-PCV	7/1	11/6
Mean greatest diameter of SMH, DD	4.0 ± 1.6 (2.0–6.7)	
Mean BCVA, logMAR (range in decimal)	0.96 ± 0.65 (0.01–0.7)	1.82 ± 0.67 (LP (+)−0.6)
Mean time from onset to initial PPV, days	9.3 ± 4.6 (4–17)	45.9 ± 40.0 (13–142) excluding 4 eyes with unknown duration

Data are expressed as number of patients/eyes or mean ± standard deviation. SMH: submacular hemorrhage, VH: vitreous hemorrhage, IOL: intraocular lens, CNV: choroidal neovascularization, PCV: polypoidal choroidal vasculopathy, RAP: retinal angiomatous proliferation, tAMD: typical age-related macular degeneration, DD: disc diameter, BCVA: best-corrected visual acuity, LP: light perception, PPV: pars plana vitrectomy.

**Table 2:** Additional procedures during initial PPV and treatments before and after initial PPV.

	SMH	VH
Number of eyes	8	17
Treatment before initial PPV		
** **Anti-VEGF	2	8
** **PDT	0	3
** **PC	0	1
Additional procedure during PPV		
** **PEA-IOL	6	11
** **tPA	8	5
** **IVB	3	3
** **Tamponade SF6/SO/air	7/0/1	2/2/0
After initial PPV		
** **Anti-VEGF	6	7
** **PDT	0	3
** **PC	0	3
** **Additional PPV	2	2

SMH: submacular hemorrhage, VH: vitreous hemorrhage, PPV: pars plana vitrectomy, VEGF: vascular endothelial growth factor, PDT: photodynamic therapy, PC: photocoagulation, PEA: phacoemulsification-aspiration, IOL: intraocular lens, tPA: tissue plasminogen activator, IVB: intravitreal bevacizumab, SO: silicone oil.

**Table 3:** Anti-VEGF therapy before and after PPV.

	SMH	VH
Number of eyes	8	17
Before initial PPV		
** **No. eyes with anti-VEGF therapy	2 (25.0%)	8 (47.1%)
** **No. of injections	6.0 ± 4.2 (3–9)	5.5 ± 5.4 (1–16)^*∗*^
** **Time from initial anti-VEGF to PPV, months	32.5 ± 10.6 (25–40)	21.6 ± 15.3 (4–49)^*∗*^
** **Duration of anti-VEGF therapy, months	8.1 ± 7.5 (2.8–13.3)	4.0 ± 2.9 (1.2–8.0)^*∗*^
After initial PPV		
** **No. of eyes with anti-VEGF therapy	6 (75.0%)	7 (41.2%)
** **No. of injections	8.5 ± 4.9 (1–15)	5.0 ± 3.1 (1–8)
** **Time from PPV to final visit, months	32.6 ± 6.7 (24–45)	53.7 ± 21.4 (25–96)
** **Duration of anti-VEGF therapy, months	10.3 ± 17.1 (2.3–45.0)	14.7 ± 10.7 (3.1–32.0)
** **Time from PPV to anti-VEGF initiation, months	6.5 ± 9.3 (1–25)	8.4 ± 11.1 (1–32)
** **Dry macula after PPV	4 (50.0%)	12 (70.6%)

Data are expressed in number of patients (percentage) or mean ± standard deviation (range). ^*∗*^ unknown in two eyes.

**Table 4:** Clinical characters, treatments, and outcomes of individual cases analyzed in this study.

Baseline						During PPV		Before PPV						After PPV							Final condition	BCVA			
Case	Gender	Age (Y)	Onset-PPV (day)	CNV subtype	SMHGD (DD)	Cataract surgery	tPA anti-VEGF	Anti-VEGF	IV (times)	Method	Other treatments	Initial treatment to PPV (M)	Anti-VEGF interval (M)	Anti-VEGF	PPV to IV (M)	IV (times)	Method	Other treatments	PPV to final (M)	Anti-VEGF interval (M)	Macula dry or wet	Before (decimal)	After 1 month	Final	Before-final (log MAR)
SMH																									
1	F	70	13	PCV	4.1	PEAIOL	tPA + IVB	—	—	—	—	—	—	IVA	2	10	LD1PRN→TAE→Fix3M	—	24	2.40	ΔduringTAE	0.07	0.2	0.4	0.76
2	M	75	6	tAMD	3.5	PEAIOL	tPA	—	—	—	—	—	—	IVA	7	7	LD1PRN→Fix3M→TAE	PPV (MH)	32	4.57	ΔduringTAE	0.05	0.3	0.2	0.60
3	M	83	4	PCV	3.1	PEAIOL	tPA	IVA	3	LD3PRN	—	40	13.33	IVA	2	6	LDITAE3M→PRN	—	28	4.67	〇	0.03	0.4	0.1	0.52
4	M	64	4	PCV	2.5	PEAIOL	tPA	—	—	—	—	—	—	IVA	1	15	LD3PRN	—	38	2.56	×	0.4	0.12	1	0.40
5	M	76	12	PCV	4.3	PEAIOL	tPA + IVB	—	—	—	—	—	—	IVA	2	12	LDITAE	—	28	2.33	×	0.5	0.6	0.6	0.08
6	F	85	17	PCV	2.0	PEAIOL	tPA	—	—	—	—	—	—	—	—	—	—	—	35	—	◎	0.01	0.04	0.01	0.00
7	F	48	9	PCV	5.7	Lens preservation	tPA + IVB	—	—	—	—	—	—	—	—	—	—	—	30	—	◎	0.7	0.5	0.4	−0.24
8	F	89	9	PCV	6.7	Already IOL	tPA	IVR IVA	9	LD1PRN	—	25	2.78	IVA	25	1	LD1PRN	PPV (PVR)	45	45	〇	0.15	0.1	0.02	−0.88

VH																									
9	F	72	14	PCV	—	PEAIOL	—	—	—	—	—	—	—	IVA	5	3	LD1PRN	PDT before anti-VEGF	65	21.67	〇	0.01	0.6	1.2	2.08
10	F	89	53	PCV	—	Already IOL	—	—	—	—	PDT	49	—	—	—	—	—	—	50	—	◎	LP (+)	0.3	0.15	1.88
11	M	78	62	PCV	—	Already IOL	IVB	IVA	Unknown	Unknown	—	Unknown	—	—	—	—	—	PC	36	—	◎	HM	CF	0.6	1.63
12	M	74	39	PCV	—	PEAIOL	IVB	—	—	—	—	—	—	—	—	—	—	—	96	—	◎	LP (+)	0.02	0.02	1.00
13	M	68	24	tAMD	—	PEAIOL	tPA	IVR IVA	3	LD3PRN	—	24	8.00	—	—	—	—	PPV (VH), PPV + SB (RD)	29	—	◎	CF	0.06	0.1	0.85
14	M	81	21	PCV	—	PEAIOL	tPA	IVA	3	LD3PRN	—	6	2.00	IVA	1	8	LD1PRN	PDT × 3 after anti-VEGF	37	4.68	Δrest SMH	0.01	0.15	0.05	0.70
15	M	83	102	RAP	—	Already IOL	—	IVB	1	LD1PRN	—	4	4.00	—	—	—	—	—	76	—	◎	0.01	0.02	0.04	0.60
16	M	86	19	PCV	—	PEAIOL	—	—	—	—	—	—	—	IVA	32	1	LD1PRN	—	32	32.00	×	0.12	0.6	0.4	0.52
17	M	67	18	PCV	—	PEAIOL	—	—	—	—	PDT	28	—	IVR	10	8	LD1PRN	PDT after anti-VEGF	73	9.13	〇	0.04	0.1	0.1	0.40
18	F	79	Unknown	PCV	—	PEAIOL	—	IVR	Unknown	Unknown	PDT	Unknown	—	IVA	9	5	LD1PRN	PC	52	10.40	×	LP (+)	0.01	HM	0.40
19	M	82	75	PCV	—	Already IOL	tPA + IVB	—	—	—	—	—	—	—	—	—	—	—	28	—	◎	HM	0.02	0.01	0.30
20	M	83	Unknown	RAP	—	Already IOL	—	IVR	4	LD3PRN	—	28	7.00	—	—	—	—	—	84	—	◎	HM	0.03	0.01	0.28
21	M	75	15	tAMD	—	PEAIOL	tPA	—	—	—	PC	Unknown	—	—	—	—	—	PPV (SOremove)	69	—	◎	0.08	0.03	0.15	0.27
22	F	82	13	PCV	—	PEAIOL	—	—	—	—	—	—	—	—	—	—	—	PC	65	—	◎	0.6	1.2	1	0.22
23	M	67	142	tAMD	—	PEAIOL	—	IVA	16	LD3PRN	—	27	1.69	IVA	1	8	LD1PRN→Fix3-4M	—	25	3.13	×	0.02	CF	HM	−0.59
24	M	67	Unknown	PCV	—	PEAIOL	tPA	IVA	6	LD3PRN	—	7	1.17	IVA	1	2	LDIPRN	—	44	22.00	〇	0.04	0.05	0.01	-0.60
25	F	80	Unknown	tAMD	—	Already IOL	—	—	—	—	—	—	—	—	—	—	—	—	51	—	ΔSO + SMH	CF	CF	LP (−)	−1.15

PPV: pars plana vitrectomy; BCVA: best-corrected visual acuity; Y: year; CNV: choroidal neovascularization; SMH: submacular hemorrhage; GD: greatest diameter; DD: disc diameter; tPA: tissue plasminogen activator; VEGF: vascular endothelial growth factor; IV: intravitreal injection; M: months; logMAR: logarithm of minimal angle resolution; VH: vitreous hemorrhage; F: female; M: male; PCV: polypoidal choroidal vasculopathy; tAMD: typical age-related macular degeneration; RAP: retinal angiomatous proliferation; IVB: intravitreal bevacizumab; SO: silicone oil; IVA: intravitreal aflibercept; IVR: intravitreal ranibizumab; LD: loading dose; PRN: pro le nata; TAE: treat and extend; MH: macular hole; PVR: proliferative vitreous retinopathy; PDT: photodynamic therapy; PC: photocoagulation; SB: scleral buckling; RD: retinal detachment; ME: macular edema; LP: light perception; HM: hand motion; CF: counting finger. Condition at final follow-up: ◎: dry macula without anti-VEGF after PPV; 〇: dry macula after anti-VEGF discontinued because of reduced activity, △: no fluid but proactive treatment ongoing or wet but anti-VEGF discontinued because of no additional benefit, ×: wet and anti-VEGF therapy ongoing.

## Data Availability

The datasets during and/or analyzed during the current study available from the corresponding author on reasonable request.
